# Nicotine replacement therapy use among smokers and ex-smokers: associated attitudes and beliefs: a qualitative study

**DOI:** 10.1186/1471-2458-14-1311

**Published:** 2014-12-22

**Authors:** Kabay Silla, Emma Beard, Lion Shahab

**Affiliations:** Department of Epidemiology and Public Health, University College London, London, WC1E 6BT UK

**Keywords:** Harm reduction, Smoking, NRT, Long-term use

## Abstract

**Background:**

Smokers who are unwilling or unable to quit smoking may benefit from using nicotine replacement therapy (NRT) for harm reduction. This may include the partial or complete substitution of cigarettes with NRT. A taxonomy of the characteristics of those using NRT for harm reduction would be helpful in tailoring advice and treatment. Although attempts to categorize those using NRT for harm reduction have been made, these have largely been based on quantitative data. In order to provide further in-depth exploration of views, beliefs and experiences, the current study probed issues surrounding NRT and harm reduction qualitatively to better understand barriers and facilitators to this approach.

**Methods:**

Three groups of participants (n = 15) were recruited from a student sample: current smokers with a history of NRT use, smokers without a history of NRT use, and ex-smokers with a history of NRT use. Participants were asked about their demographic characteristics, smoking behaviours, intention and perceived ability to quit smoking, awareness and use of NRT, beliefs about the health consequences of using NRT, and the safety and efficacy of NRT, using semi-structured telephone interviews.

**Results:**

Twenty-four themes were identified; these themes were clustered into three main issues of cross-cutting themes: attitudes towards smoking and motivation to quit; smoking reduction and quit attempts; and beliefs, use and concerns about NRT. Those with a history of NRT use were more motivated and engaged with the quitting process than non-users. However, irrespective of smoking status and past NRT use, all participants showed misperceptions about NRT, such as the health consequences associated with NRT use.

**Conclusions:**

NRT users are more motivated to quit smoking than non-users and are more likely to employ techniques to assist their cessation attempts. The majority of smokers have misperceptions regarding the safety and efficacy of NRT which may act as a barrier to its usage.

**Electronic supplementary material:**

The online version of this article (doi:10.1186/1471-2458-14-1311) contains supplementary material, which is available to authorized users.

## Background

Harm reduction aims to lessen the negative effects from smoking without complete cessation [1]. This can be done in a number of ways, including the partial or complete substitution of cigarettes with safer nicotine replacement therapy products (NRT). Partial replacement includes using NRT as a means to achieve smoking reduction (SR; cutting down the number of cigarettes smoked) or for periods of temporary abstinence (TA; using NRT during periods when smoking is prohibited). In contrast, complete substitution involves replacing *all* cigarettes with NRT often to prevent relapse. Substantial evidence supports the use of harm reduction. In both clinical trials and population-based studies, the partial substitution of cigarettes with NRT increases smokers’ propensity to stop smoking and in certain circumstances results in significant reductions in cigarette consumption (which may be associated with reduced immediate harm) [1, 2]. Studies have also demonstrated that the long-term use of NRT following smoking cessation helps to prevent relapse to smoking [3].

Globally, there are over 1.3 billion smokers [4] and smoking has been estimated to be responsible for the death of 12% of all adults aged 30 years and older [5]. In 2012, a national survey estimated cigarette smoking prevalence in England at 20 per cent (using a sample of 21,330) and quitting numbers (i.e. quitters per year) were 6.2 per cent of 4,726 participants. In 2014, the same survey found that smoking prevalence was 18.5% of 15, 206 respondents and cessation had risen to 7.5 per cent of a sample of 3,147 [6]. However, despite changes in regulation, particularly in the UK where NRT is licensed for harm reduction purposes and recommended as a tobacco control policy [7]; few smokers use NRT long-term to prevent relapse or to help reduce their cigarette intake [1, 8]. Consequently, there is a need to identify ways in which we can encourage smokers, attempting to cut down, to use NRT (the most effective method) and better still, encourage smokers who are unable or unwilling to quit nicotine, to substitute their cigarettes completely with safer NRT products. A suitable first step is to develop a typology of smokers who use and do not use NRT for partial or complete substitution in order to tailor interventions to encourage smokers to use NRT and aid their cessation attempts. Previous quantitative surveys have shown that the partial substitution of cigarettes with NRT is more common among married smokers with high nicotine dependence and is associated with age, ethnicity and social-economic status [9]. Moreover, long-term NRT use appears to be more common among older, female smokers of higher socio-economic status who are more dependent on cigarettes [10].

These findings are insightful as they reveal the socio-demographic profile of smokers attempting harm reduction with NRT. However, it is not feasible to develop a full taxonomy through the use of quantitative data alone; response categories tend to be of closed format or fixed. Although this inflexibility allows for easy comparison across participants, it restricts the richness of information obtained. Qualitative research can fill this gap.

The following study investigates the beliefs and views regarding NRT and tobacco use, of ex-smokers with a history of NRT use, smokers with a history of NRT use, and smokers who have never used medicinal nicotine products. We aim to determine the characteristics of those who engage in NRT use and to identify the barriers and facilitators of the use of medicinal nicotine in order to inform harm reduction policies and interventions aimed at smokers who struggle to stop smoking.

## Methods

### Design and procedure

Three groups of participants were recruited via an email advertisement to all UCL students: current smokers with a history of NRT use, smokers without a history of NRT use, and ex-smokers with a history of NRT use. These three groups were chosen in order to provide an insight into the various stages of the decision processes involved in using or not using NRT and reasons for continued use and associated success or failure to stop. We chose a student population as it was easily accessible in the recruitment phase, providing a suitable mix of NRT naïve and NRT experienced smokers and ex-smokers. Additionally, within the current and ex-smokers with a history of NRT use groups, there were a mixture of people who were and were not using NRT at the time. Participants had to be 18+ years of age, speak English and be in good health. Information regarding NHS stop smoking services was offered to participants after the interview, and they were compensated for their time with a £10 high street voucher. Participants were provided with information sheets about the purpose of the study and informed consent was obtained from all participants. Ethical approval was granted by the UCL Research Ethics Committee. This study complies with the RATS guidelines.

A semi-structured interview design was used to provide guidance, but also to obtain an in-depth understanding of the issues. The telephone interviews were conducted between May and June 2012. The interviews lasted no longer than 30 minutes and were recorded via a telephone recorder. A current smoker was defined as a person who reported having smoked at least 100 cigarettes in their lifetime and who currently smokes every day or on most days. Additionally, as three months therapy is the standard time frame given to smokers who chose to use NRT for harm reduction in the UK [7] and due to the fact that it may be difficult to find participants that have been using NRT for a longer period of time, the long-term use of NRT was defined to be 3 months and longer, which allows for the inclusion of more participants in the target pool from which selection took place.

### Participants

134 smokers and ex-smokers respond to the advertisement of whom 105 could not be contacted. Of the 29 that were left, 2 failed to meet the inclusion criteria. The first 15 participants (5 from each group) that responded and met the inclusion criteria took part in the study as a sample size of 15 was judged adequate to reach saturation [11].

#### Measures

Topic guides for the interviews were developed from existing literature, PRIME theory [12] and based on findings from a previous quantitative study [10]. The survey instrument included questions on the following subjects: demographic characteristics, smoking behaviours, intention and perceived ability to quit smoking, awareness and use of NRT, beliefs about the health consequences of using medicinal nicotine, and the safety and efficacy of NRT.

Three interview schedules were created to meet the needs of each group of participants and covered 1) current smoking behaviour; 2) beliefs, views and experience of smoking; 3) their current goals; 4) views about NRT; 5) whether they were using NRT and why; 6) which products they were using; 7) their views on the harmfulness and effectiveness of NRT; 8) their knowledge of the current regulations of NRT; 9) other forms of treatment to quit smoking. The interview schedules included questions such as; “What are your views, beliefs and experience of smoking?”; “What are your views about NRT?”; “What are your thoughts about long-term NRT use?”; and “Have you tried anything else to quit smoking?”

#### Data analysis

Interviews were recorded and transcribed verbatim. Transcripts were analysed by KS using framework analysis [13] to identify recurring themes and patterns within the data. Framework analysis allowed us to examine differences between the three groups and look for explanations for any differences identified. This was done using five key stages. This first involved becoming familiar with the interview transcripts by rereading in order to identify emerging key themes and sub-themes. Following this, concepts were then indexed to corresponding themes and applied to the framework. The final stage of the process involved analysing the key characteristics by making comparisons and explanations from the data.

Internal validity was attained by using ‘deviant case analysis’ and the ‘constant comparative’ method. A number of transcripts were randomly selected and read by another coder (EB) in order to establish external validity [14]. This coder verified that the transcripts were analysed reliably and that that the information included supported the studies’ main findings.

## Results

Of the sample of 15 participants, 9 were male and 6 were female, with an average age of 27 years (*SD* ± 7.9). Seven identified themselves as White British, three White, two Chinese, two White Chinese and one White American. The respondents reported smoking for an average of 11 years (*SD* ± 7.5). Current smokers also stated that they have a cigarette consumption of 11 cigarettes per day (*SD* ± 5.9). Ex-smokers revealed that their previous cigarette consumption averaged at 22 cigarettes per day (*SD* ± 4.5).

Those with a history of NRT use (i.e. ex-smokers and current smokers that have used or are currently using NRT; [mean = 27, SD = 2.17]; [mean = 32; SD = 10.98] respectively), were older than smokers who have not used NRT (mean = 22, SD = 2.17). In regards to cigarette consumption, those with a history of NRT use generally smoked more cigarettes daily (ex-smokers with a history of NRT use [mean = 22, SD = 4.47]; smokers with a history of NRT use [mean = 15, SD = 5.55]) than smokers without a history of NRT use (mean = 8, SD = 4.34). Long-term NRT use (lasting beyond the standard 3 months of therapy) was found amongst 60% of the ex-smokers and 10% of the current smokers with a history of NRT. Ex-smokers and current smokers with a history of NRT use stated that they have tried the nicotine gum, patches and lozenges.

Twenty-four themes were identified; eighteen of which were deemed relevant to the proposed aims of the study: to determine the characteristics of those who engage in the use of NRT; and to identify the barriers and facilitators of the use of medicinal nicotine. These themes have been clustered into 3 main issues of cross-cutting themes (Table 1): attitudes towards smoking and motivation to quit; smoking reduction and quit attempts; and beliefs, use and concerns about NRT. These issues are discussed below. The main themes and sub-themes that may play a role in whether harm reduction is employed are detailed in Table 2. Additional themes can be found in Additional file 1: Table S1.

**Table 1:** **Factors that may influence whether smoking reduction or cessation occurs, and which may encourage NRT usage**

Issue	Factors	Similarities	Differences	Associated sub-themes ^*^
1. Attitudes towards smoking and motivation to quit	Negative attitudes, emotions, and experience of smoking	All groups		1.1, 3.1, 4.1
	Factors that encourage quitting	ExNRT, sNRT		3.2
			sNRT	3.3
			ExNRT	3.4
	The role of motivation and control to quit/cut down	ExNRT, sNRT		7.4
			ExNRT	6.1
			SNRT	6.2
			sNRT	6.3
	Smoking cessation as a future goal	All groups		8.1
2. Smoking reduction and quit attempts	Abrupt quitting and cutting down	All groups		9.1
		ExNRT, SNRT		10.2
			ExNRT	9.2
			SNRT	10.3
			sNRT	10.1
	Behavioural methods to assist cessation	ExNRT, SNRT		11.3
			ExNRT	11.1, 11.2
	Stop smoking services		SNRT	24.1, 24.2
3. Beliefs, use and concerns about NRT	Factors that will encourage the use of NRT	ExNRT, SNRT		12.1, 12.4
			SNRT	12.5
			sNRT	15.1, 15.2
	Incorrect/under-use of NRT	ExNRT, SNRT		16.2
	Short-term use of NRT	ExNRT, SNRT		17.1
	Long-term use of NRT		ExNRT	17.2
	Knowledge about NRT	All groups		18.1
	Concerns about NRT	All groups		19.1, 19.2, 19.4
		ExNRT, SNRT		19.5
		SNRT, sNRT		19.3
	Recommend NRT and other forms of therapy	All groups		22.1, 23.2

Ex-NRT = Ex-smoker NRT; SNRT = Smoker NRT sNRT = Smoker no NRT; ^*^Detailed in Table 2.

**Table 2:** **Main themes that may influence whether smokers engage in harm reduction**

Theme	Sub-theme
1. Views on smoking	1.1 Hostility towards smoking
3. Reasons to quit smoking	3.1 To improve health
	3.2 Social stigma
	3.3 Saving money
	3.4 Personal
4. Emotions connected with smoking initiation	4.1 Regret starting
6. Beliefs that influence quit attempts	6.1 Control of nicotine dependence
	6.2 Motivation and willpower
	6.3 Confidence
7. Difficulties quitting/cutting down	7.4 Lack of motivation
8. Goals	8.1 Smoking cessation
9. Methods used to quit smoking	9.1 Abrupt
	9.2 Cut down first
10. Methods of cutting down	10.1 Not smoking as many cigarettes
	10.2 Not smoking in the morning
	10.3 Smoking only part of the cigarette
11. Non-medical aids to quit smoking	11.1 Distraction
	11.2 Motivation
	11.3 Exercising
12. Reasons for using NRT	12.1 To aid quit attempt
	12.4 To help with cravings
	12.5 Rid of habit
15. Factors to encourage use of NRT	15.1 Free samples
	15.2 Aid quit attempt
16. Use of NRT	16.2 Incorrect use/underuse
17. Length of NRT use	17.1 Short-term
	17.2 Long-term
18. Knowledge about NRT	18.1 Current regulations
19. Concerns about NRT	19.1 Addiction
	19.2 Harmful when used long-term
	19.3 Effective
	19.4 Health consequences
	19.5 Delays quitting process
22. Helpfulness of NRT	22.1 Recommend
23. Recommendation of cessation aids	23.2 Other forms of therapy
24. Stop smoking services	24.1 Advantages
	24.2 Disadvantages

Themes and sub-themes included focus on the associated themes in Table 2.

### Issue 1: attitudes towards smoking and motivation to quit

Respondents from each group expressed negative attitudes towards smoking and stated that they regret having started smoking (Table 2) primarily due to the addictiveness of cigarettes and the health consequences of smoking. Ex-smokers and smokers with a history of NRT use generally indicated that these reasons were the motivating factors for wanting to quit:

‘[Regret starting because] I haven’t been able to stop. And because yeah it’s expensive and addicting and disgusting and deadly yeah… I would quit if I could, I’m trying.’ (GZ, 29 year old female, smoker NRT).

Although smokers without a history of NRT use also shared these views and stated that they regret smoking because of their inability to quit, a few stated that they are not yet ready to quit. The perception of needing to want to quit (which was generally viewed as a prerequisite to the quitting process) may influence the use of NRT as smokers may not want to use NRT unless they are ready to quit smoking. This suggests that smokers do not understand that the use of NRT is potentially valuable in helping them strengthen the want to quit smoking.

‘If the person’s a bit sort of ambivalent about whether they want to quit or not, then NRT is not going to be that much help. If they’re ready to kind of just quit anyway then yeah NRT is going to aid them to do that. But it just depends on how much the person wants to quit.’ (MW, 22 year old male, smoker NRT).

Furthermore, even though both groups of current smokers revealed that they had plans to quit smoking (Table 2), these plans were often vague and set sometime in the distant future. Respondents failed to detail whether sub-goals were set; whether they wanted to quit abruptly, or cut down gradually. Only one smoker with a history of NRT use indicated that they would use NRT as an aid. This demonstrates that transforming intention into behaviour is quite difficult as intentions do not always trigger a behavioural response.

### Issue 2: smoking reduction and quit attempts

A few respondents used a variety of techniques to gradually cut down cigarette consumption (Table 2) such as reducing the number of cigarettes smoked, (which was used by smokers who have no history of NRT use) but this was not found to be very effective. Not smoking in the morning was tried by respondents with a history of NRT use in order to cut down cigarette consumption.

The most common method used for previous quit attempts by all groups was abrupt quitting (Table 2), which involved going ‘cold turkey’ for as long as possible. Again, respondents who used this method believed that willpower and motivation are the most important factors involved when quitting and this determination influences the success of the quit attempt.

‘I didn’t use anything, any aids or whatever, I just stopped smoking. Erm… I think because I just had the right motivation.’ (CS, 25 year old female, smoker no NRT).

Additionally, ex-smokers stated that they had high levels of motivation and employed distraction techniques to support their quit attempts. Exercising was used by ex-smokers and smokers with a history of NRT use in order to keep them busy and feel healthier which in turn encouraged them to abstain from smoking. These non-medical aids were only employed by those with a history of NRT use (Table 2). Additionally, only a minority of respondents had used the stop smoking services suggesting that individuals are either unaware of their availability, or do not believe that they have a need for it. Smokers with a history of NRT use credited the stop smoking service for providing information regarding withdrawal symptoms prior to the experience of withdrawal. However, this service was criticised by smokers with a history of NRT use for being over-reliant on NRT for smoking cessation.

### Issue 3: beliefs, use and concerns about NRT

Respondents with a history of NRT use indicated that they are aware of the function of NRT as they decided to use medicinal nicotine in order to aid their quit attempt; help with cravings and to modify some behavioural patterns and situational cues that became associated with smoking. For example, smoking after a meal or whilst drinking alcohol.

‘I think I thought that if I used, like any of the NRT things and when I have that moment [of craving], and I felt like I couldn’t overpower it, I would be able to if I had some nicotine [NRT].’ (AT, 32 year old female, ex-smoker NRT).

Also, the cost of NRT played a big role in whether smokers invested in them (a view shared by both ex-smokers and smokers who have never used NRT). Non-users indicated that they would be encouraged to use NRT if they were able to collect free samples to test how effective the products are, whereas other said that they would use medicinal nicotine to assist their quit attempt if they knew that it would increase their chances (Table 2).

‘I guess what can be done is NRT should probably be given free samples to smokers when they try first so that they will actually understand how it works and whether it is effective for them.’ (GL, 22 year old male, smoker no NRT).

Amongst NRT users, ex-smokers reported using it for longer periods of time than smokers (Table 2). Ex-smokers revealed a determination to quit smoking, and used NRT for such a long time in order to prevent relapse. However, ex and current smokers with a history of NRT use were worried about transferring their nicotine addiction to NRT, which resulted in short-term, incorrect or under-use use of the products. That is, NRT users sometimes failed to adhere to the specific instructions given regarding how each NRT product should be administered, the frequency of use and the dosage. This demonstrates that smokers have concerns and misperceptions about medicinal nicotine with regards to addictiveness, believing that it may act as a gateway back into smoking. Each group of respondents had mixed feelings about whether using NRT long-term is harmful but generally believed that using NRT is less harmful than smoking cigarettes.

‘Compared to smoking it’s probably virtually no harm compared to smoking… I’d be very surprised if they were approved and sold on the national health system if they were going to do that much harm to you.’ (MW, 22 year old male, smoker NRT).

In terms of the effectiveness, current smokers believed that NRT is efficacious to a certain extent and may only work for some individuals. Smokers and ex-smokers thought that using NRT may have health consequences even though they were not sure what to attribute these health consequences to. NRT users also believed that using medicinal nicotine delays the quitting process as their withdrawal symptoms lasted longer. Despite this, the majority of ex-smokers and smokers with a history of NRT use (Table 2) would recommend NRT but would set conditions on their recommendation based on the length of time that NRT should be used for in order to prevent dependence. This suggests that although the majority of respondents believed that use of NRT is safer than smoking, they still held misconceptions about NRT that may act as a barrier towards its use. All groups of respondents also suggested that they would recommend both NRT and other forms of therapy, indicating that smokers would like additional support when attempting to quit.

## Discussion

This study aimed to determine the barriers and facilitators of the use of medicinal nicotine; and to determine the characteristics of those who engage in the use of NRT; in order to find ways to encourage smokers to engage in harm reduction.

All groups of smokers were found to hold negative opinions about smoking. Smokers with a history of NRT use appeared motivated to want and attempt to quit whereas those without a history of NRT use seemed to lack this motivation. Those using NRT long-term were characterised as being highly motivated to quit smoking, engaged in various methods to assist their quit attempts such as exercising and employing distraction techniques, and revealed that they used medicinal nicotine as it greatly assisted their cessation goal and helped to maintain their motivation to quit.

The current study also identified a number of barriers and facilitators of the use of medicinal nicotine. One barrier was the belief that smokers did not require NRT to quit smoking. Previous studies have shown that smokers often believe that they will be successful with smoking cessation without assistance [15, 16]. Indeed, “cold turkey” is the most common method utilised by both smokers and ex-smokers in their current and previous quit attempts [17]. Unfortunately, it is also the least successful method [18]. One way to increase the use of NRT for harm reduction may therefore be to modify their control beliefs, i.e. inform smokers that an effective method of harm reduction and smoking cessation involves a combination of behavioural and medicinal support, to tackle both the behavioural and psychological aspects of the addiction [19].

Additionally, another barrier of the use of medicinal nicotine for harm reduction was the cost of NRT. Non-users reported that several factors will encourage them to use NRT, including being able to collect free samples to test the effectiveness of the products. However, University students are a select group and may be more price-sensitive to the general population [20]. As NRT for harm reduction is generally obtained over the counter, rather than on prescription [21], it appears that income and the cost of NRT are factors that affect the usage of NRT [10]. Previous studies have suggested that the usage of NRT may increase if smokers are provided with free NRT products [22, 23], and having NRT programmes wherein smokers are able to find the NRT product that is most effective for them through trialling [24]. These strategies may reduce the social inequalities found in the use of NRT for harm reduction [25].

Interestingly, misperceptions regarding the safety and efficacy of NRT persist across all groups, and this acts as a barrier towards the use of NRT for harm reduction. This is in line with previous findings on the use of NRT for smoking reduction and/or temporary abstinence e.g. [26]. Smokers and ex-smokers were found to hold substantial concerns about medicinal nicotine which may act as a barrier towards its use in the long-term, or incorrect or under-use [27, 28] among those who opt for complete substitution of NRT with cigarettes. Many reported being concerned about becoming addicted to NRT and were worried about the health consequences associated with NRT. These are commonly held beliefs by many smokers and ex-smokers [29, 30]. This is despite the fact that becoming addicted to medicinal nicotine is rare [31, 32] and that there are few if any health implications [33].

In relation to engaging in harm reduction, all groups of smokers regretted having started smoking for various reasons including health effects, and the addictiveness of cigarettes [34]. However, those who have never used NRT indicated that they were less likely to have these concerns. Previous studies have noted that concerns about health effects are associated with help seeking behaviour [35]. Consequently, NRT use for harm reduction may be more likely among those with health effect concerns. This relates to our previous finding [10] showing that smokers concurrently using medicinal nicotine are more motivated to quit smoking than non-NRT users. The use of NRT not only helps individuals form the motivation and ‘want’ to engage in a quit attempt [36], but intervention studies have also shown that it helps to reduce smoking in smokers with no interest in quitting [37].

This study has a number of limitations. Firstly, as with all qualitative work, the findings cannot be generalised to the wider population as this study focused on a particular sub-group within the population. Secondly, although saturation was attained as no new themes emerged from the last few participants; further themes may have emerged if a larger sample was used. Thirdly, it is probable that the interpretation of qualitative data can be subject to bias and error. Despite this, every effort has been made to eliminate the occurrence of this by adopting quality assurance techniques to ensure that the interpretation of the responses accurately reflects the data. Fourthly, even within the context of qualitative research providing an in-depth understanding of smokers and ex-smokers attitudes and beliefs regarding smoking and the use of NRT, the findings may be limited by the interpretations made and not having been able to address all the relevant issues. However, the findings provide information on preliminary observations that can be further examined using objective measures.

## Conclusions

This study attempts to determine the characteristics of NRT users and to identify the barriers and facilitators of NRT use for harm reduction. This study reveals that the use of NRT is characterized by individuals who are motivated to quit and willing to engage in the process of quitting by employing medical and non-medical aids to assist their quit attempts. NRT users tended to be more concerned about the health effects of smoking, and this acted as a motivating factor in the use of NRT. We find a number of barriers to the general use of NRT, ranging from misconceptions regarding its safety and efficacy to the cost of NRT, which may also undermine the use of NRT for harm reduction. Health professionals need to be made aware of the need to educate users of these products, not only in terms of their proper application but also regarding the safety and efficacy within the context of harm reduction and eventual smoking cessation. In order to encourage smokers to use NRT for both cessation and particularly harm reduction purposes, future interventions should aim to reduce the cost of NRT especially for those who are unwilling or unable to quit smoking.

## Electronic supplementary material

Additional file 1: **Themes associated with beliefs about smoking, quit attempts and cutting down cigarette consumption and the beliefs, use and concerns about NRT.** (DOCX 44 KB)

## Abbreviations

MHRA: Medicines and Healthcare products Regulatory Agency
NRT: Nicotine Replacement therapy
SR: Smoking reduction
TA: Temporary abstinence.
