# Supplementary material for: Nicotine replacement therapy use among smokers and ex-smokers: associated attitudes and beliefs: a qualitative study
Source: BMC Public Health. 2014 Dec 22;14:1311. doi: 10.1186/1471-2458-14-1311 (PMC4364650; doi:10.1186/1471-2458-14-1311)
Supplement: Supplementary file 1 — Additional file 1: Themes associated with beliefs about smoking, quit attempts and cutting down cigarette consumption and the beliefs, use and concerns about NRT. (DOCX 44 KB) [file 12889_2014_7439_MOESM1_ESM.docx]

**Supplementary Table 1: Themes associated with beliefs about smoking, quit attempts and cutting down cigarette consumption and the beliefs, use and concerns about NRT**

| **Theme** | **Sub-Theme** | **Status** | **Example** |
| --- | --- | --- | --- |
| 1. Views on smoking | 1.1 Hostility towards smoking | Ex-Smokers NRT | ‘I understand it [why people smoke] but I’m really, I think I’m quite against it. Like I really don’t think parents should be allowed to smoke around children. Like I think it’s wrong that I grew up in a smoking household and you know that affects, you know your health and your future attitudes towards it.’ (LL, 20 year old female, ex-smoker).  ‘I think it’s a good thing that we don’t smoke in restaurants anymore but that’s because it ruins the food than anything else… I don’t like it when I see people smoking around their kids but other than that it don’t bother me really.’ (AT, 32 year old female, ex-smoker).  ‘I’m a little bit weary of err… of becoming addicted to it again and I don’t want to… I don’t mind if people smoke around me… I think it’s a horrible product because I think it will kill you if you continue.’ (TW, 31 year old male, ex-smoker). |
|  |  | Smokers NRT | ‘Extremely anti, in fact, I think that it should be illegal… Because it’s addicting and it kills people. I mean if anything is a drug it’s totally a drug. I mean there’s, I think there’s nothing good about it.’ (GZ, 29 year old female, smoker NRT).  ‘It is quite antisocial… just because fewer and fewer people I know smoke and it’s unpleasant for people around you.’ (DR, 23 year old male, smoker NRT).  ‘I would say negative [attitude towards smoking]. I don’t like smoking…I associate cigarettes with all the bad things that go with it like the lack of freedom, like being tired.’ (NL, 38 year old male, smoker NRT). |
|  |  | Smoker no NRT | ‘I think it’s bad and I know it’s bad for you and I always think I should stop smoking. Erm… you know I just haven’t.’ (CS, 25 year old female, smoker no NRT). |
| 2. Reasons for enjoying smoking | 2.1 Habit | Ex-smoker NRT | ‘It’s just really sociable and like it becomes a part of your everyday life. Like you have a cigarette break and whatever, you go to the shop and say hello to the shopkeeper and yeah so that’s the hardest part I guess.’ (LL, 20 year old female, ex-smoker).  ‘I like the way that it feels like you’re taking a break from everything because you’re having a cigarette and it’s just… that’s it you’re going to chill out, get away from everything.’ (AT, 32 year old female, ex-smoker). |
|  | 2.2 Social | Ex-smoker NRT | ‘It’s quite sociable and I know it’s always called an anti-social habit but I met some of my best friends because we were all smokers.’ (AT, 32 year old female, ex-smoker). |
|  |  | Smoker no NRT | ‘My friends are mainly smokers so the influence is very high so I guess I’m still ok with it.’ (GL, 22 year old male, smoker no NRT).  ‘…just find it a nice sort of thing to do especially in the evenings. Erm, sort of when other people are around.’ (VW, 19 year old female, smoker no NRT). |
|  | 2.3 Personal | Ex-smoker NRT | ‘I like the way it tastes and I like the way it smells, all the things that you’re not meant to but yeah.’ (AT, 32 year old female, ex-smoker).  ‘I’ve always liked the taste, I liked the idea of err… you know taking a break… I’ve always enjoyed smoking when going out and you know having wine, beer and stuff, I enjoy it quite a lot.’ (TW, 31 year old male, ex-smoker). |
|  |  | Smoker NRT | ‘There’s times where I really enjoy it… it’s a drug, I suppose it’s err… you enjoy taking the drug that our body craves.’ (SL, 48 year old male, smoker NRT).  ‘I like the err… the sort of the peace and quiet of having a cigarette by yourself. I find it helps with the stress.’ (DR, 23 year old male, smoker NRT). |
|  | 2.4 Mood | Ex-smoker NRT | ‘It increased my spirit... it was quite pleasurable. Yes. I mean when I was quite sleepy, or when I was frustrated basically yeah, smoking could err give me confidence and err spirit yeah.’ (BL, 23 year old male, ex-smoker NRT). |
| 3. Reasons to quit smoking | 3.1 To improve health | Ex-smoker NRT | ‘I would wake up in the morning I had chest pains and err… and a bad cough.’ (TW, 31 year old male, ex-smoker).  ‘I think it makes you more susceptible to colds and I just sort of… it was that that made me stop… And your lung capacity is, I think, I found is greatly reduced if you’re smoking heavily, and even at all. Yes, I mean, very short-term health things like that.’ (JR, 30 year old male, ex-smoker NRT). |
|  |  | Smoker NRT | ‘I feel like I’ve smoked for quite a long time now and it’s going to start, you know, having major effects on my health if I don’t stop soon.’ (DR, 23 year old male, smoker NRT).  ‘I’ve been smoking for a very long time now, my health is not good, my stamina is not good. It’s not that I’m completely unfit, it’s just err… I’m certainly not fit at all so…’ (SL, 48 year old male, smoker NRT). |
|  |  | Smoker no NRT | ‘Mostly it’s unhealthy and it seems like, because I don’t smoke constantly all day, it seems like something that wouldn’t be that hard to give up.’ (CS, 25 year old female, smoker no NRT).  ‘I think I got ill before with tonsillitis and coughing and it’s not related directly with smoking, you know, it does make you think if I didn’t smoke maybe I wouldn’t be so ill.’ (KH, 22 year old female, smoker no NRT). |
|  | 3.2 Social stigma | Ex-smoker NRT | ‘Not a lot of people that I became friends with [at university] smoked so it was a bit weird, like I was the only one smoking.’ (LL, 20 year old female, ex-smoker). |
|  |  | Smoker no NRT | ‘Most of my friends don’t smoke anymore and, you know, I always feel a little bit guilty about it.’ (CS, 25 year old female, smoker no NRT).  ‘I do not really like being a smoker because it always leaves a negative impression on people… You kind of look a bit older and also smoking cause us to take breaks and reduces our productivity.’ (GL, 22 year old male, smoker no NRT). |
|  | 3.3 Saving money | Smoker no NRT | ‘It’s expensive and although I don’t feel too dependent, I know a lot of people who are very dependent.’ (KH, 22 year old female, smoker no NRT). |
|  | 3.4 Personal | Ex-smoker NRT | ‘I always said that I was going to give up when I was either 25 or when a packet of cigarettes hit five pounds or when I was pregnant. And a packet of cigarette was well over five pounds and I was well over 25 and I’m not going to get pregnant in the next year but I thought I was probably too old to be still smoking and it cost a fortune so yeah.’ (AT, 32 year old female, ex-smoker).  ‘I’ll have a cigarette and then you feel lethargic straight after because of it like, you feel a bit dirty, the smell, and you’ll be a bit… and I kind of don’t know, it is actually a bit disgusting.’ (JR, 30 year old male, ex-smoker NRT). |
|  |  | Smoker NRT | ‘I feel like I’ve smoked for quite a long time now and it’s going to start, you know, having major effects on my health if I don’t stop soon.’ (DR, 23 year old male, smoker NRT).  ‘I’ve been smoking for a very long time now, my health is not good, my stamina is not good. It’s not that I’m completely unfit, it’s just err… I’m certainly not fit at all so…’ (SL, 48 year old male, smoker NRT). |
|  |  | Smoker no NRT | ‘Mostly it’s unhealthy and it seems like, because I don’t smoke constantly all day, it seems like something that wouldn’t be that hard to give up.’ (CS, 25 year old female, smoker no NRT).  ‘I think I got ill before with tonsillitis and coughing and it’s not related directly with smoking, you know, it does make you think if I didn’t smoke maybe I wouldn’t be so ill.’ (KH, 22 year old female, smoker no NRT). |
| 4. Emotions connected with smoking initiation | 4.1 Regret starting | Ex-smoker NRT | ‘I think I do [regret smoking] because I used to be quite good at sports… as soon as I started smoking I became really crummy at sports and I didn’t want to do anything… it [smoking] just makes you more breathless.’ (LL, 20 year old female, ex-smoker)  ‘The health that I have lost is definitely greater than the pleasure that I have gained, so I do regret.’ (BL, 23 year old male, ex-smoker NRT). |
|  |  | Smoker NRT | ‘[Regret starting because] I haven’t been able to stop. And because yeah it’s expensive and addicting and disgusting and deadly yeah… I would quit if I could, I’m trying.’ (GZ, 29 year old female, smoker NRT).  ‘Well it’s obviously very bad for your health, it costs a lot of money, err it makes you smell, it uses up an awful lot of time, waste a lot of time. That’s probably the most irritating thing, it stops you doing things, you have a cigarette before you do something, you have a cigarette after you’ve started something.’ (SL, 48 year old male, smoker NRT). |
|  |  | Smoker no NRT | ‘It just seems like it was a bad decision when I was younger and, you know, it continues to impact my life now.’ (CS, 25 year old female, smoker no NRT).  ‘I do because now I don’t sort of quite know when I’d stop and when I’d give up. Erm… so I’m annoyed with myself for starting in the first place yeah.’ (VW, 19 year old female, smoker no NRT). |
|  | 4.2 Do not regret starting | Smoker NRT | ‘I don’t regret because I know that I can change it.’ (NL, 38 year old male, smoker NRT). |
|  |  | Smoker no NRT | ‘No [don’t regret starting]. Nothing negative has come of it.’ (BT, 21 year old male, smoker no NRT). |
| 5. Emotions connected with smoking cessation | 5.1 Achievement | Ex-smoker NRT | ‘I think yeah it’s a sense of achievement because I finally quit.’ (BL, 23 year old male, ex-smoker). |
|  |  | Smoker NRT | ‘The other benefits was, that I found was a sort of pride to be able to control that [nicotine addiction].’ (NL, 38 year old male, smoker NRT). |
|  | 5.2 Worried that may become addicted again | Ex-smoker NRT | ‘…I was worried about not being able to sustain my, my, my quitting attempt.’ (TW, 31 year old male, ex-smoker). |
| 6. Beliefs that influence quit attempts | 6.1 Control of nicotine dependence | Ex-smoker NRT | ‘I think a lot of people don’t realise how addicted they are to cigarettes until they try to stop.’ (AT, 32 year old female, ex-smoker). |
|  | 6.2 Motivation and Willpower | Smoker NRT | ‘If the person’s a bit sort of ambivalent about whether they want to quit or not, then NRT is not going to be that much help. If they’re ready to kind of just quit anyway then yeah NRT is going to aid them to do that. But it just depends on how much the person wants to quit.’ (MW, 22 year old male, smoker NRT).  ‘I think what is important when you’re giving up is to really make the decision and be prepared to make the effort to make this happen.’ (NL, 38 year old male, smoker NRT). |
|  | 6.3 Self-efficacy in ability to quit | Smoker no NRT | ‘Well I think that just generally speaking I don’t really like to try and fail. So I think if I’m not pretty sure that I’m going to succeed at quitting then I probably, I’m less likely to try it.’ (CS, 25 year old female, smoker no NRT). |
| 7. Difficulties quitting/cutting down | 7.1 Alcohol | Ex-smoker NRT | ‘Sometimes when you are out with other people smoking you do have the urge, especially under the influence of alcohol.’ (JR, 30 year old male, ex-smoker NRT). |
|  | 7.2 Withdrawal Symptoms | Smoker NRT | ‘It’s very hard to focus, which means that your concentration level decrease so I found that when I’m not smoking I have low concentration because the fact I’m not smoking makes my brain work a lot slower… And the second thing is that when you give up smoking you have loads of digestive problems so that was another reason why I went back on smoking.’ (NL, 38 year old male, smoker NRT). |
|  | 7.3 Stress | Ex-smoker NRT | ‘It took over a year to actually quit… as soon as something stressful happens like our body remembers like sort of the relief you got from smoking and like that’s all you want… the relief of having that drug’ (LL, 20 year old female, ex-smoker). |
|  |  | Smoker no NRT | ‘I would go back when I’m feeling stressed or I’m in trouble. So I’ll start smoking again which… you know I can’t quit because of all that.’ (GL, 22 year old male, smoker no NRT). |
|  | 7.4 Lack of motivation | Ex-smoker NRT | ‘I hadn’t really thought it through about how I was going to do this [previous unsuccessful quit attempt] and it wasn’t really in my head that I was being really determined about it and I probably in reality didn’t really want to I just sort of felt like I should give up.’ (AT, 32 year old female, ex-smoker).  ‘Ultimately I think that you do have to have a certain degree of not wanting to smoke, and I don’t know if I had that properly enough at all [during previous unsuccessful quit attempt].’ (JR, 30 year old male, ex-smoker NRT). |
|  |  | Smoker no NRT | ‘With smoking because you don’t see the damage it’s doing its quite difficult to sort of, to sort of rationalise in your mind why it’s so bad for you if you can’t see the, you know, damage to your lungs and stuff, it’s quite difficult to kind of get that motivation together.’ (VW, 19 year old female, smoker no NRT).  ‘Even though I think about quitting, but I’m not that motivated yet. It seems like a really good idea though.’ (CS, 25 year old female, smoker no NRT). |
|  | 7.5 Mood | Ex-smoker | ‘I remember, I remember feeling a real sense of loss. Sounds ridiculous but I did. I mean I felt like err I had lost a part of myself.’ (TW, 31 year old male, ex-smoker). |
|  | 7.6 Habit (feel dependent on cigarettes) | Ex-smoker NRT | ‘I was getting nicotine withdrawal… the patches or whatever like, they could stop all of that happening but they don’t actually stop you wanting a cigarette.’ (LL, 20 year old female, ex-smoker).  ‘It’s such a habit, it’s such a part of your life, you know, I’d finish dinner and I’d want to have a cigarette where other people will want to have desert… just getting out of all those habits is quite difficult.’ (AT, 32 year old female, ex-smoker).  ‘It was more the idea that something was missing and life was a lot less fun now. And I felt a bit redundant after having had food and I felt like I was… I didn’t know when to stop a meal and I didn’t feel like I was taking any proper breaks or I didn’t know really what to do.’ (TW, 31 year old male, ex-smoker).  ‘I think the power of habit is, is very strong honestly. If you have cultivated a habit for several years it definitely, it takes several times to quit that. So I think it is normal. Yes. And eventually yes I, I quit so I think it’s a regular process.’ (BL, 23 year old male, ex-smoker NRT). |
|  |  | Smoker NRT | ‘Because you might have one or two and before you know it you’re back to where you were before so… it’s a highly addictive drug and once you get back on it it’s very easy to pull back into old habits.’ (SL, 48 year old male, smoker NRT). |
|  |  | Smoker no NRT | ‘The hardest part was just the habit of smoking…a lot of times I’ll smoke when I drive or when I’m waiting on the street for a friend or something. So when I find myself in those situations and I’m not smoking I notice that.’ (CS, 25 year old female, smoker no NRT).  ‘Because of the addiction I guess. Because erm… most of the time when you start smoking it’s also a habit. I’m really used to smoking after meals so it’s a very strong habit which I’m finding it hard to kick.’ (GL, 22 year old male, smoker no NRT). |
| 8. Goals | 8.1 Smoking cessation | Ex-smoker NRT | ‘Well I’ve given up in the past for a year and then I went back to smoking. So I guess my immediate goal was to get past that year which is about now in fact so yeah.’ (AT, 32 year old female, ex-smoker). |
|  |  | Smoker NRT | ‘I would like to quit either before I’m 30 or if I, well obviously if I have children.’ (GZ, 29 year old female, smoker NRT).  ‘…plans to quit at some point but vague plans.’ (DR, 23 year old male, smoker NRT).  ‘…as of the 1^st^ of June I will definitely give up and I will use some NRT.’ (NL, 38 year old male, smoker NRT). |
|  |  | Smoker no NRT | ‘I’m pretty confident that I’ll quit in the next 5 years… I guess I should rephrase and say I hope I quit in the next 5 years. There’s no specific plan or reason.’ (CS, 25 year old female, smoker no NRT).  ‘I am pretty confident that I will be able to quit but erm I guess not exactly right now. Probably two years later… on average I guess I’m going to get married in two years. Err… this pressure would motivate me to quit.’ (GL, 22 year old male, smoker no NRT).  ‘I intend to quit… because I’m aware the longer I smoke for the more likely I would have problems later on in life… in 5 years time I definitely don’t want to be smoking.’ (KH, 22 year old female, smoker no NRT). |
|  | 8.2 Social smoking | Smoker NRT | ‘I want to become a social smoker so basically, but that doesn’t work very well for me. But ideally, yeah I would say my goal is to either completely give up or to be a social smoker and I’ll smoke a few times a year.’ (NL, 38 year old male, smoker NRT). |
| 9. Methods used to quit smoking | 9.1 Abrupt | Ex-smoker NRT | ‘Just not smoking. I know it sounds really silly but it’s willpower when it comes down to it… it’s a question of saying I’m not going to light that cigarette when someone offers it to you or, and just reminding yourself that you really do want to stop.’ (AT, 32 year old female, ex-smoker). |
|  |  | Smoker NRT | ‘Well it was going cold turkey really but I read the Allen Carr’s quitting smoking book which I thought was quite good.’ (DR, 23 year old male, smoker NRT).  ‘I decide that I’m not going to smoke for the whole day. Then I use that for the whole day and then if I can go on another day then I go on for another day. Sometimes I go with a goal, like I’d be like I’m going to quit smoking by then and then I go until a certain day and then I see if I can go until then. But I don’t really follow their instructions really.’ (GZ, 29 year old female, smoker NRT). |
|  |  | Smoker no NRT | ‘I didn’t use anything, any aids or whatever, I just stopped smoking. Erm… I think because I just had the right motivation.’ (CS, 25 year old female, smoker no NRT). |
|  | 9.2 Cut down first | Ex-smoker NRT | ‘I just cut down and then eventually after cutting down until like one day I just managed to stop.’ ‘The easiest way was just kind of cutting down to like one a day or like rolling smaller cigarettes.’ (LL, 20 year old female, ex-smoker). |
| 10. Methods of cutting down | 10.1 Not smoking as many cigarettes | Smoker no NRT | ‘I usually tried to reduce the number of cigarettes… Most of the time I would just reduce the number of pack. I don’t think it’s very effective. Probably going cold turkey will be better I guess.’ (GL, 22 year old male, smoker no NRT).  ‘I just cut down, kind of gave myself a max to have each day and then just stopped.’ (BT, 21 year old male, smoker no NRT). |
|  | 10.2 Not smoking in the morning | Ex-smoker NRT | ‘I think maybe one of the things that was most successful when I gave up this time around is that I was really determined that one of the first things that I was going to give up was smoking in the mornings… I think once I broke that habit as well that made it kind of easier to not fee like I need the next cigarette at 11 o clock and the next one at lunchtime and, you know, because I hadn’t had one that day so.’ (AT, 32 year old female, ex-smoker). |
|  |  | Smoker NRT | ‘Because I’m err someone that smokes all day. I get up, have a coffee then have a cigarette so cutting down those first ones is a very good way of stopping that habit.’ (SL, 48 year old male, smoker NRT). |
|  | 10.3 Smoking only part of the cigarette | Smoker NRT | ‘Yeah I would say maybe a third of the time. A lot of the time I would start smoking it and then I’m just like why am I doing this, I hate myself and stuff like that. Yeah that’s quite frequent actually.’ (GZ, 29 year old female, smoker NRT).  ‘Quite often at the moment. I think that because I mean it’s sort of, trying to improve my health, so I would say almost every cigarette I wouldn’t finish. Because I’m a bit disgusted as well so I would not finish it.’ (NL, 38 year old male, smoker NRT). |
| 11 Non-medical aids for smoking cessation | 11.1 Distraction | Ex-smoker NRT | ‘Like I used to do like some horse riding and stuff like that and I found that if I was working and doing that all day then I didn’t think about a cigarette all day and it was really useful to help with quitting.’ (LL, 20 year old female, ex-smoker). |
|  | 11.2 Motivation | Ex-smoker NRT | ‘I read the Allen Carr book…I think it was probably not necessarily the book that made me quit, but it was the fact that I had taken all that time to do something that was positive towards quitting… it was more just the, this kind of complete commitment to the fact that I had decided to give up so much so that I wanted to read a book about it.’ (AT, 32 year old female, ex-smoker). |
|  | 11.3 Exercising | Ex-smoker NRT | ‘Probably go cycling]… And swimming. I think swimming is very useful because… swimming is a good exercise of our lungs so I think for smokers swimming is… it could be effective to quit the err smoking.’ (BL, 23 year old male, ex-smoker NRT). |
|  |  | Smoker NRT | ‘I started exercising more which encourages you to quit smoking just because you feel healthier.’ (DR, 23 year old male, smoker NRT).  ‘Exercise is good, just keep you busy.’ (SL, 48 year old male, smoker NRT). |
| 12. Reasons for using NRT | 12.1 To aid quit attempt | Ex-smoker NRT | ‘I was really determined to give up smoking and I thought the more help I can get the better.’ (TW, 31 year old male, ex-smoker).  ‘I think it’s very effective… I think it improves your chances significantly. I mean at least for me. I could never have done it without, especially the lozenges.’ (TW, 31 year old male, ex-smoker). |
|  |  | Smoker NRT | ‘I was thinking about quitting at the time and I also wanted to see how effective they were.’ (MW, 22 year old male, smoker NRT).  ‘I thought it might make it easier. I thought that you would be able to quit without any withdrawal symptoms.’ (DR, 23 year old male, smoker NRT).  ‘To help me give up smoking... it’s only an extra tool. Err… it’s the will power that’s the main, the main one.’ (SL, 48 year old male, smoker NRT).  ‘[I used NRT because] I had to stop smoking. It gets in the way of my life completely. I mean if I just don’t smoke then I get really unproductive and I turn mean.’ (GZ, 29 year old female, smoker NRT). |
|  | 12.2 Past use | Ex-smoker NRT | ‘I had tried NRT before, I mean when flying overseas and stuff like that, I had chewed nicotine chewing gum. So I knew that it had some sort of an effect and then it was about combating the physical craving of smoking.’ (TW, 31 year old male, ex-smoker). |
|  | 12.3 Recommended | Ex-smoker NRT | ‘My father is a heavy smokers and he suggested me to use that.’ (BL, 23 year old male, ex-smoker NRT). |
|  | 12.4 To help with cravings | Ex-smoker NRT | ‘I think I thought that if I used, like any of the NRT things and when I have that moment [of craving], and I felt like I couldn’t overpower it, I would be able to if I had some nicotine.’ (AT, 32 year old female, ex-smoker NRT). |
|  |  | Smoker NRT | ‘If I really had a craving I don’t buy cigarettes, I’d buy the chewing gum instead.’ (NL, 38 year old male, smoker NRT).  ‘They’re going to help my managing the nicotine addiction for the first days and reduce the withdrawal symptoms for the first days.’ (NL, 38 year old male, smoker NRT). |
|  | 12.5 Rid of habit | Smoker NRT | ‘I think it is a good way to cut down, I think it’s a good way to get rid of the habit, I don’t, I mean I used to smoke before and after every meal, every hour, at work sometimes blah blahblah. I don’t have any habits associated with smoking anymore.’ (GZ, 29 year old female, smoker NRT). |
| 13. Reasons for not using NRT | 13.1Cost | Ex-smoker NRT | ‘What’s the point in stopping spending money on cigarettes if you’re going to spend like maybe almost as much on the nicotine replacement?’ (LL, 20 year old female, ex-smoker). |
|  |  | Smoker no NRT | ‘It cost money to actually buy NRT and it can be very costly as well so I do not start using it… because NRT cost money, so many smokers will rather buy the cigarettes than to spend on NRT.’ (GL, 22 year old male, smoker no NRT).  ‘I would have to kind of look at how expensive they are. Erm… that would be quite a big factor for me, to see how, you know, if I can afford it. If not, then kind of go cold turkey.’ (VW, 19 year old female, smoker no NRT). |
|  | 13.2 Not ready to quit | Smoker no NRT | ‘Erm… basically because I don’t want to quit yet.’ (VW, 19 year old female, smoker no NRT).  ‘I’ve never wanted to stop yet.’ (KH, 22 year old female, smoker no NRT). |
|  | 13.3 Problems with the product | Ex-smoker NRT | ‘A lot of it isn’t very pleasant, like the gums, the lozenges aren’t very nice to take and like the patches, they’re ok like but they’re a bit annoying, you know, peel off and wear on your arm and they’re a bit unsightly and stuff.’ (LL, 20 year old female, ex-smoker). |
|  | 13.4 No need psychologically | Smoker no NRT | ‘I think because I only smoke maybe 3 or 4 cigarettes a day… So if I’m, if I miss smoking for like a day or two I don’t actually really notice. Erm… so I, I, don’t think the NRT would really help me personally. (CS, 25 year old female, smoker no NRT).  ‘I didn’t feel like, I wouldn’t be averse to it, I just didn’t feel like I needed it.’ (BT, 21 year old male, smoker no NRT). |
|  | 13.5 Other methods | Ex-smoker NRT | ‘Some people believe that it’s better to go cold turkey and just see it as a bit of err… I don’t know…maybe it’s slightly harsh to say that it’s sign of weakness that you have to use it… maybe some people find that it’s just another substitute for smoking and don’t want to be reminded of the fact that they are addicted to something.’ (TW, 31 year old male, ex-smoker). |
|  | 13.6 Habit | Smoker no NRT | ‘It doesn’t replace the habit and like the act of smoking. So whilst you, if you quit and you really suffer from nicotine cravings, I think it helps with that, but I think it doesn’t help when you do activities that you normally associate with smoking and you’re not smoking.’ (CS, 25 year old female, smoker no NRT). |
| 14. Reasons for stopping the use of NRT | 14.1 Achieved goal | Ex-smoker NRT | ‘I think because I have achieved my purposes. I quit smoking yes. I don’t want the NRT to be a new addiction yeah so yeah I stop it.’ (BL, 23 year old male, ex-smoker NRT). |
|  | 14.2 Cost | Ex-smoker NRT | ‘You buy a pack of patches and it sets you back how much like twenty quid or something.’ (LL, 20 year old female, ex-smoker).  ‘I think it is quite difficult to use long-term because it’s expensive.’ (AT, 32 year old female, ex-smoker).  ‘It must be better and cheaper just to buy a pack of polos and just eat those instead of the lozenges. It was quite expensive I remember, I think I could spend up to, I don’t know, a tenner a week on NRT and I thought that was a bit too much.’ (TW, 31 year old male, ex-smoker). |
|  | 14.3 Substitution | Ex-smoker NRT | ‘I think in terms of the gum because I have another replacement, I can use a normal gum, so it wasn’t hard [to stop using NRT] actually.’ (BL, 23 year old male, ex-smoker NRT). |
|  |  | Smoker NRT | ‘I would replace the NRT with proper chewing gums.’ (NL, 38 year old male, smoker NRT). |
|  | 14.4 Started smoking again | Smoker NRT | ‘[I stopped using NRT because] I got a new job and I started smoking again because it was stressful and because a lot of my work colleagues were smoking as well so.’ (DR, 23 year old male, smoker NRT). |
| 15. Factors to encourage use of NRT | 15.1 Free samples | Smoker no NRT | ‘I guess what can be done is NRT should probably be given free samples to smokers when they try first so that they will actually understand how it works and whether it is effective for them.’ (GL, 22 year old male, smoker no NRT).  ‘I guess if it was a free a free thing I could do to help me stop smoking.’ (KH, 22 year old female, smoker no NRT). |
|  | 15.2 Aid quit attempt | Smoker no NRT | ‘[Would be encouraged to use NRT] Just to kind of ease the transition I guess.’ (VW, 19 year old female, smoker no NRT).  ‘I think it’s something I would definitely consider if I felt that I couldn’t stop on my own.’ (KH, 22 year old female, smoker no NRT).  ‘If I read somewhere that it increases your chances and if I was finding it difficult. That’s about it. I’d only ever use it if I couldn’t quit naturally.’ (BT, 21 year old male, smoker no NRT). |
| 16. Use of NRT | 16.1 Concurrent use | Ex-smoker NRT | ‘I’d go into school with a smoking patch on and then use the gum and just have a fag anyway. So it was stupid.’ (LL, 20 year old female, ex-smoker). |
|  | 16.2 Incorrect use/under-use | Ex-smoker NRT | ‘I guess I didn’t chew the gum in the way intended which is kind of constantly chew it like you do the patches sort of thing, I would just chew it when I had the craving.’ (AT, 32 year old female, ex-smoker).  ‘I’ll just have one when I feel like it. Which to be honest is almost most of the time, when I’m awake I have some sort of nicotine product in my mouth which is probably not… you lose, I think you lose control of it.’ (JR, 30 year old male, ex-smoker NRT). |
|  |  | Smoker NRT | ‘The gum you’re suppose to like chew it until you taste it and then like store it somewhere in your mouth and then it like feeds into you like, through this like some thin membrane or something in your mouth. But I don’t use it that way so I don’t really know. I just chew it but erm… yeah.’ (GZ, 29 year old female, smoker NRT).  ‘I basically cut them in quarters to avoid having too much of an intake of the replacement at the same time.’ (NL, 38 year old male, smoker NRT). |
|  | 16.3Correct use | Smoker NRT | ‘It was like a plaster, you just patch it onto your skin. Erm… every x number of hours, I mean every 4 hours or every 10 hours or something like that… yeah I did, yeah [use according to the instructions].’ (DR, 23 year old male, smoker NRT). |
| 17. Length of use | 17.1 Short-term | Ex-smoker NRT | ‘About 3 weeks probably… I think just part of my motivation was that I wanted to give up smoking is not being tied to something and not being addicted to something… it was very much about that craving moment and giving myself the nicotine in the split second that I need it.’ (AT, 32 year old female, ex-smoker). |
|  |  | Smoker NRT | ‘A few days, a week or two… afterwards I decided that I didn’t really think they were that good and I wasn’t particularly ready to stop smoking anyway. So I just decided to stop doing that and carry on as I was before.’ (MW, 22 year old male, smoker NRT).  ‘Every time it’s for a short period, periods of time… I would use it for 2 days and I would decrease the amount over a week and I would not chew then after the week.’ (NL, 38 year old male, smoker NRT). |
|  | 17.2 Long-term | Ex-smoker NRT | ‘I used it for about 8 or 9 months… I drew quite fond of it actually. I liked the err the mint lozenges and I err sort of developed a taste for them that you know… I was scared that if I had gone off too quickly, that I would be tempted to smoke again.’ (TW, 31 year old male, ex-smoker).  I’ve used it for probably seven months… it wasn’t a very long [smoking] history. Probably that’s why I have just used NRT for several months and I eventually quit smoking.’ (BL, 23 year old male, ex-smoker NRT).  ‘Well I mean constantly, I mean on and off it’s been over 10 years. Constantly I would say it would be 8 years.’ (JR, 30 year old male, ex-smoker NRT). |
|  | 17.3 Temporary abstinence | Ex-smoker NRT | ‘I don’t reckon I used it for more than a month at a time… if I was going on holiday with like my dad who didn’t smoke, or didn’t know I smoked, I’d like use it then but only really as an alternative to smoking, not to quit sort of thing.’ (LL, 20 year old female, ex-smoker). |
| 18. Knowledge about NRT | 18.1 Current regulations | Ex-Smoker NRT | ‘No I don’t [have any knowledge about the regulations of NRT].’ (AT, 32 year old female, ex-smoker NRT). |
|  |  | Smoker NRT | ‘No [I don’t have any knowledge about the regulations of NRT], I assumed that they can’t be sold to minors, but that’s what I assume, I don’t know.’ (NL, 38 year old male, smoker NRT). |
|  |  | Smoker no NRT | ‘No, no idea.’ [In response to whether she has any knowledge about the regulations of NRT]. (KH, 22 year old female, smoker no NRT). |
| 19. Concerns about NRT | 19.1 Addiction | Ex-smoker NRT | ‘A real thing for me was that I didn’t want to be addicted anymore and I think while you are still addicted there’s always a risk that you’ll go back to the thing that’s really pleasurable, and the nicotine replacement therapy is never going to be as pleasurable as cigarette so while you’ve still got that addiction I think there is a risk that you’ll go back to smoking.’ (AT, 32 year old female, ex-smoker).  ‘I have no doubt that they’re just as addictive as erm… as cigarettes. But whether or not it’s bad to have an addiction I don’t really know.’ (TW, 31 year old male, ex-smoker).  ‘Essentially I’ve quit smoking but I haven’t quit nicotine. Erm… still thoroughly addicted to nicotine…I was smoking 20 cigarettes a day which I think is about 1.5mg, it’s 30mg of nicotine I was potentially absorbing when I was smoking and, but since I’ve been on nicotine replacement, I think that’s gone right up and at its peak I think I was on about 60 or more, so I’d be chewing over 115 four mg tablets or chewing gum a day. So I actually think it’s easier to become more addicted on NRT than I think it is cigarettes.’ (JR, 30 year old male, ex-smoker NRT). |
|  |  | Smoker NRT | ‘Erm… it works but then as soon as it’s done then it doesn’t really work anymore because you’re still sort of addicted to nicotine.’ (GZ, 29 year old female, smoker NRT).  ‘You know I met someone the other day that gave up 7 years ago and is still using the gum. I don’t think that’s good… because you’re still dependent on the drug that you were dependent on when you were actually smoking. You know being dependent on any drug is not good.’ (SL, 48 year old male, smoker NRT).  ‘I think that the current treatments are not adapted to some smokers because they, some treatments would make people more addicted to nicotine than they were before… I think that they are overdosed, that they don’t not necessarily cater for light smokers.’ (NL, 38 year old male, smoker NRT). |
|  |  | Smoker no NRT | ‘Erm… honestly I don’t know much about NRT at this moment but there is still nicotine involved and I believe in some ways it’s still a little harmful to a certain extent… probably like addiction because you are still dependent on nicotine. So this addiction is somewhat harmful, probably not in a health way but in another way.’ (GL, 22 year old male, smoker no NRT). |
|  | 19.2 Harmful when used long-term | Ex-smoker NRT | I don’t know if it’s harmful because I don’t really know the science behind… But I can imagine that it’s not great for you and erm… like, I don’t know, I think it’s, people like telling themselves that they’re, you know, overcoming their addiction when really they’re still feeding it but just like in a different way. So I think it’s not very good to use it long-term.’ (LL, 20 year old female, ex-smoker).  ‘It stops you smoking so I don’t think it’s harmful, I’m not sure it does you any good necessarily because like I say you’ve still got an addiction there and that’s something that you really need to kick. But erm, yeah I don’t think it’s harmful, I don’t know of anything it does that, it’s not really the nicotine in the cigarette that’s really bad for you, it’s all the other sh*t.’ (AT, 32 year old female, ex-smoker). |
|  |  | Smoker NRT | ‘Compared to smoking it’s probably virtually no harm compared to smoking… I’d be very surprised if they were approved and sold on the national health system if they were going to do that much harm to you.’ (MW, 22 year old male, smoker NRT).  ‘It can’t be as harmful as cigarettes I think.’ (NL, 38 year old male, smoker NRT). |
|  |  | Smoker no NRT | ‘I don’t think I know enough about it to know if it would be harmful. I’d assume it would be less harmful than actually smoking but that’s just my gut feeling, I don’t actually know.’ (CS, 25 year old female, smoker no NRT).  ‘No not at all, I’m not aware that it is. I don’t know that much about it.’ (BT, 21 year old male, smoker no NRT).  ‘I think 3 months sounds like a long time to use nicotine replacement… It just doesn’t sound very good for you to be, you know, having nicotine put through your skin or chewing it in the gum, erm… sort of other, yeah, it just, they just sound like too long. I think maybe a month to be using something. If you’re trying to quit, I think a month to use a replacement is kind of sufficient.’ (VW, 19 year old female, smoker no NRT). |
|  | 19.3 Effective | Smoker NRT | ‘I think for some people yeah, but I think it’s probably more to do with the mental aspect, about how you frame it in your own mind. I think your attitude going into quitting smoking is the most important thing and if using NRT helps with the attitude then it’s probably good.’ (DR, 23 year old male, smoker NRT). |
|  |  | Smoker no NRT | ‘To a certain extent yes because they replace the nicotine that we require. But sometimes smoking is psychological. Many may not be so dependent on the nicotine itself. Sometimes they just have the habit of doing it.’ (GL, 22 year old male, smoker no NRT).  ‘I should think they’re very effective. Erm, I mean they must be because they have such a large consumer market.’ (VW, 19 year old female, smoker no NRT). |
|  | 19.4 Health consequences | Ex-smoker NRT | ‘I guess at the time I didn’t think about it because I wasn’t doing a science degree but now I think that obviously any method of taking in a drug like can have adverse effects… But at the time I thought oh well it’s got to be better than smoking.’ (LL, 20 year old female, ex-smoker).  ‘Erm I don’t really know of any. I think to be honest I never looked into that which is silly isn’t it? But I guess because the health consequences of smoking are so bad that I assumed that anything else would be better.’ (AT, 32 year old female, ex-smoker).  ‘Whether or not specifically it harms you I’m not sure. I mean I don’t know if nicotine is bad for you to be honest. I mean I, of course I should… I, I’m under the impression that it isn’t so bad for you as smoking.’ (TW, 31 year old male, ex-smoker). |
|  |  | Smoker NRT | ‘I can’t imagine that they’re as bad as smoking.’ (GZ, 29 year old female, smoker NRT).  ‘You buy them in pharmacies so, you kind of assume that, well certainly in the long run as well, whatever the side effects are they’re probably, they’re probably less than carrying on smoking. You know, in terms of you health so, I guess whatever effects kind of would seem a lot smaller in comparison to the health effects of smoking.’ (DR, 23 year old male, smoker NRT).  ‘That people could get throat cancer or some issues with the tongue because of chewing NRT for too long. I don’t know but that’s something that I’ve heard.’ (NL, 38 year old male, smoker NRT). |
|  |  | Smoker no NRT | ‘No because… not really thought of the health consequences of it, ever thought about it.’ (GL, 22 year old male, smoker no NRT). |
|  | 19.5 Delays quitting process | Ex-smoker NRT | ‘Nicotine replacement therapy sort of delays that point at which you’re going to have to say to yourself no I’m not going to have one. So I don’t really think it makes it any easier, it just prolongs it.’ (LL, 20 year old female, ex-smoker). |
|  |  | Smoker NRT | ‘I think when I tried it I find it was actually more difficult because when I’ve tried to quit smoking other ways, you get sort of 3 days of bad withdrawal symptoms and then after that it starts to get easier whereas NRT just drags out that period for a longer period of time.’ (DR, 23 year old male, smoker NRT).  ‘NRT just postpones these deadlines, the point where you’re going to have like 48 hours where it’s going to be more difficult so but the NRT delays that a bit.’ (NL, 38 year old male, smoker NRT). |
| 20. Benefits of products | 20.1 Nicotine gum | Ex-smoker NRT | ‘I have just used for seven months and I have quit smoking.’ (BL, 23 year old male, ex-smoker NRT).  ‘You’ve got the chewing gum you can stick it in your mouth and you can actually physically recognise and feel that its got something in it which is the nicotine… It’s a mental connection with your supply of drug.’ (JR, 30 year old male, ex-smoker NRT). |
|  | 20.2 Nicotine patch | Ex-smoker NRT | ‘I found the patches helpful sort of to wear like maybe at school or something. And in terms of not making you like have that sort of agitated, angry feeling when you don’t have a cigarette.’ (LL, 20 year old female, ex-smoker). |
|  | 20.3 Nicotine lozenges | Ex-smoker NRT | ‘I drew quite fond of it actually, I liked the err, the err the mint lozenges and I err sort of developed a taste for them.’ (TW, 31 year old male, ex-smoker NRT). |
| 21. Side effects & disadvantages of products | 21.1 Nicotine gum | Ex-smoker NRT | ‘The gum was ok but It tasted really rank and erm… yeah so you don’t really want it in your mouth.’ (LL, 20 year old female smoker).  ‘The chewing gum I think is absolutely vile so I stopped using it quite quickly. It hits the back of your throat and it just makes you want to throw up.’ (AT, 32 year old female, ex-smoker).  ‘They make your mouth and lips sore sometimes. If you have one stuck on the side of your mouth it can make your skin go a bit funny.’ (JR, 30 year old male, ex-smoker NRT). |
|  |  | Smoker NRT | ‘Erm… gum I loath, I didn’t want to chew gum, I can’t bear, I have used it but it upsets me to chew it because it’s just disgusting.’ (SL, 48 year old male, smoker NRT) |
|  | 21.2 Nicotine Patch | Ex-smoker NRT | ‘I haven’t had much success with patches when I tried them cause I find patches there’s no connecting… you put a patch on and it doesn’t really work instantly.’ (JR, 30 year old male, ex-smoker NRT).  ‘My skin would get sort of irritated when I had the patches even though I didn’t have the patches on the same, on the same spot everyday.’ (TW, 31 year old male, ex-smoker). |
|  |  | Smoker NRT | ‘Some brands [of the patch] give me like really weird blisters.’ (GZ, 29 year old female, smoker NRT).  ‘It made your skin itch sometimes but nothing serious.’ (DR, 23 year old male, smoker NRT).  ‘The patches dreadful for your skin, horrible the skin reaction, you get these big red blotches… that are very itchy that you, you know especially at night time you itch.’ (SL, 48 year old male, smoker NRT). |
|  | 21.3 Nicotine lozenges | Ex-smoker NRT | ‘There is sort of a fair, small degree of problem your mouth has to put up with taking the lozenges.’ (JR, 30 year old male, ex-smoker NRT).  ‘They get white stuff all around your lips… it’s not a pleasant feeling in your mouth just cause it taste all peppery.’ (LL, 20 year old female, ex-smoker). |
|  |  | Smoker NRT | ‘It makes your mouth very chalky, then you get little white things all over your mouth.’ (GZ, 29 year old female, smoker NRT). |
| 22. Helpfulness of NRT | 22.1 Recommend | Ex-smoker NRT | ‘I guess I would maybe recommend people give it a try for maybe like the first week or the first couple of weeks if like, if they’re really heavy smokers. Because like the idea of not having a cigarette every 5 minutes could be quite weird. But then like once you’ve actually gone a day or a couple of days without having a cigarette and you sort of like you know what it feels like, then I guess I’d recommend stopping or cutting down or something because like using it long-term or something is not really helping and you just need to like get to that point where you body doesn’t have any nicotine in it anymore and then it’s all like psychological from there.’ (LL, 20 year old female, ex-smoker).  ‘Yeah, I really would [recommend the use of NRT to smokers]. But I would also, I would also say that maybe after about half a year that you should also may start thinking about how to get rid of the habit, in hindsight. I think you can, I think you can get yourself off it quicker than you think you can. You don’t want it to develop to become another addiction, another sort of thing you have to do so.’ (TW, 31 year old male, ex-smoker).  ‘I mean based only on my own experience, I would say that I would not advise it if they want to stop the intake of nicotine but I would advise it if they want to stop smoking.’ (JR, 30 year old male, ex-smoker NRT). |
|  |  | Smoker NRT | ‘Yeah of course. I think no matter how bad it is for you, I think it’s better for you.’ (GZ, 29 year old female, smoker NRT).  ‘Because its there to help. Rather than, you know, if someone’s going to either smoke or give up just on willpower alone, I would recommend using the thing. I think it would help.’ (MW, 22 year old male, smoker NRT).  ‘[Would recommend] To use it as something, as a kind of aid… you really need to use will power to stop completely so.’ (SL, 48 year old male, smoker NRT). |
|  |  | Smoker no NRT | ‘If I find that it’s able to make me stop and if err… it reduces my craving, I will definitely introduce it to everyone.’ (GL, 22 year old male, smoker no NRT). |
|  | 22.2 Ineffective | Ex-smoker NRT | ‘Most people that I know who have used nicotine replacement therapy… use it for however long for like weeks or months you know maybe even years and like either never come off it or need to start smoking again. I don’t know anyone that’s successfully quit because of it.’ (LL, 20 year old female, ex-smoker)  ‘It didn’t work for me at all I have to say... I was getting like the drug that was keeping me addicted to it but I wasn’t having the enjoyment of smoking. So it just made me want to smoke even more because I was like yeah nicotine is great, smoking is even better.’ (AT, 32 year old female, ex-smoker).  ‘When I finally stopped, it didn’t really help the prospect that one would never have a cigarette again in ones whole life.’ (TW, 31 year old male, ex-smoker). |
|  |  | Smoker NRT | ‘It doesn’t actually erm… make you give up smoking (inaudible)… it’s mainly the willpower that makes you give up smoking.’ (SL, 48 year old male, smoker NRT) |
|  | 22.3 Too weak | Ex-smoker NRT | ‘I never managed to cut down on the patches. Like I can use the full strength ones… then as soon as I used the smaller one I felt like, I don’t know, I felt like it wasn’t working as much so that didn’t really help.’ (LL, 20 year old female, ex-smoker). |
| 23. Recommendation of cessation aids | 23.1 NRT | Ex-smoker NRT | ‘I wouldn’t particularly [recommend NRT] no, because it didn’t work for me so.’ (AT, 32 year old female, ex-smoker). |
|  |  | Smoker NRT | ‘The only one that I would really recommend to anyone else that is helpful for me is the err… nicotine minis, the little erm… pills that you put in your mouth, under your lip.’ (SL, 48 year old male, smoker NRT). |
|  | 23.3 Combination of both | Smoker NRT | ‘Yeah sure [recommend other forms of therapy in combination with NRT], I think anything that helps you give up, but I would say it is mind over matter in many ways and that is the only way to do it.’ (SL, 48 year old male, smoker NRT). |
| 24. Stop smoking services | 24.1 Advantages | Smoker NRT | ‘They were able to tell what symptoms you were going to go through when giving up smoking… And I thought that knowing in advance what was going to happen was quite reassuring and helping building the willpower.’ (NL, 38 year old male, smoker NRT). |
|  | 24.2 Disadvantages | Smoker NRT | ‘I thought that they focusing too much on NRT… I didn’t think it was good because I thought that they were giving the wrong advice since I think that NRTs are harmful in the long-term’ (NL, 38 year old male, smoker NRT).  ‘They don’t help in managing the withdrawal symptoms. So I think that’s something where some action there would help.’ (NL, 38 year old male, smoker NRT). |
|  | 24.3 Scared about going | Ex-smoker NRT | ‘It’s just really like, I don’t know, like daunting. Like you have to book an appointment and then they tell you why you should stop smoking or something and yeah it seems a bit, it’s a bit like too much effort.’ I booked a smoking cessation appointment once but I never went … I guess I just thought that my heart wasn’t really in it and I just didn’t really, really want to quit. And it was at like 8 o clock in the evening and I just couldn’t be bothered.’ (LL, 20 year old female, ex-smoker). |
|  | 24.4 No need for them | Ex-smoker NRT | ‘No reason at all, I just thought that I could probably manage it by myself.’ (AT, 32 year old female, ex-smoker).  ‘I don’t honestly think that there is anything that they can tell me. I don’t think that they have any useful advice, that’s why I didn’t go.’ (JR, 30 year old male, ex-smoker NRT). |
|  |  | Smoker NRT | ‘When I did want to quit I just went and got the patches. I didn’t think that I needed to go to any kind of group, lessons or seminars or whatever it is.’ (MW, 22 year old male, smoker NRT)  ‘Personally I think addiction is a very habitual thing and it’s err, it’s, it’s I presume for me, not for anyone else, I don’t think their experiences will actually help me.’ (SL, 48 year old male, smoker NRT). |
|  | 24.5 Didn’t know they were available | Ex-smoker NRT | ‘Erm I guess I didn’t really know what was there, you know, what was available. Which, where to go for advice. And I always… I don’t know, I don’t mean to sound like I don’t believe in advice groups and stuff but I think quitting smoking is a very personal thing, and I didn’t really want somebody to tell me what to do or… I think it’s something you have to do for yourself, well I had to do with myself and not get other people involved too much.’ (TW, 31 year old male, ex-smoker). |
|  | 24.6 Smoking Pack | Ex-Smoker NRT | ‘I guess it was quite useful… but erm like to a certain extent like I knew all the things that they were saying. Like I knew like erm it was really bad for my health and my taste buds and everything so… I don’t know, I didn’t feel like I needed a reward system for myself to do it but I guess that other people would find that useful.’ (LL, 20 year old female, ex-smoker). |
